# Computational identification and analysis of novel sugarcane microRNAs

**DOI:** 10.1186/1471-2164-13-290

**Published:** 2012-07-02

**Authors:** Flávia Thiebaut, Clícia Grativol, Mariana Carnavale-Bottino, Cristian Antonio Rojas, Milos Tanurdzic, Laurent Farinelli, Robert A Martienssen, Adriana Silva Hemerly, Paulo Cavalcanti Gomes Ferreira

**Affiliations:** 1 Laboratorio de Biologia Molecular de Plantas, Instituto de Bioquímica Médica, Universidade Federal do Rio de Janeiro, Rua Rodolpho Paulo Rocco s/n°, CCS, Bl.B-33A, Cidade Universitária 21941-590, Rio de Janeiro, RJ, Brazil; 2 Universidade Federal da Integração Latino-Americana, Av. Tancredo Neves, 6731, Bl.4, 85867-970, Foz do Iguaçu, PR, Brazil; 3 Cold Spring Harbor Laboratory, 1 Bungtown RD, Cold Spring Harbor, NY, 11724, USA; 4 School of Biological Sciences, The University of Queensland, St Lucia, QLD 4072, Australia; 5 Fasteris SA, 1228-Plan-les-Ouates, Switzerland

**Keywords:** Small RNA, Biotic stress, Abiotic stress, Deep sequencing

## Abstract

**Background:**

MicroRNA-regulation of gene expression plays a key role in the development and response to biotic and abiotic stresses. Deep sequencing analyses accelerate the process of small RNA discovery in many plants and expand our understanding of miRNA-regulated processes. We therefore undertook small RNA sequencing of sugarcane miRNAs in order to understand their complexity and to explore their role in sugarcane biology.

**Results:**

A bioinformatics search was carried out to discover novel miRNAs that can be regulated in sugarcane plants submitted to drought and salt stresses, and under pathogen infection. By means of the presence of miRNA precursors in the related sorghum genome, we identified 623 candidates of new mature miRNAs in sugarcane. Of these, 44 were classified as high confidence miRNAs. The biological function of the new miRNAs candidates was assessed by analyzing their putative targets. The set of *bona fide* sugarcane miRNA includes those likely targeting serine/threonine kinases, Myb and zinc finger proteins. Additionally, a MADS-box transcription factor and an RPP2B protein, which act in development and disease resistant processes, could be regulated by cleavage (21-nt-species) and DNA methylation (24-nt-species), respectively.

**Conclusions:**

A large scale investigation of sRNA in sugarcane using a computational approach has identified a substantial number of new miRNAs and provides detailed genotype-tissue-culture miRNA expression profiles. Comparative analysis between monocots was valuable to clarify aspects about conservation of miRNA and their targets in a plant whose genome has not yet been sequenced. Our findings contribute to knowledge of miRNA roles in regulatory pathways in the complex, polyploidy sugarcane genome.

## Background

Small RNA (sRNA) has direct and versatile involvement as regulator of gene expression in many eukaryotes [1]. This sRNA consists of small endogeneous RNAs, 20–25 nucleotides (nt) in length, grouped into two major classes: microRNA (miRNA) and small interfering RNA (siRNA) [2]. The biosynthesis of plant miRNA starts with the transcription of MIR genes by RNA polymerase II (Pol II) into primary miRNA (pri-miRNA), generating a long-single strand RNA that has partial self-complementary, forming a characteristic hairpin structure. In Arabidopsis, this stem-loop precursor is processed by Dicer-like protein (DCL1), HYPONASTIC LEAVES 1 (HYL1) and SERRATA resulting in a duplex miRNA-miRNA*. The duplex is methylated by HUA ENHANCER 1 (HEN1) [3] and transported from the nucleus to the cytoplasm by HASTY [4]. Finally, one strand of the duplex, the mature miRNA, is inserted into a ribonucleoprotein complex, called RISC, containing the ARGONAUTE1 (AGO1) protein [5] that binds to its target messenger RNA by sequence complementarity. miRNA regulates the expression of mRNA target by mRNA target cleavage, and through translational or transcriptional repression [6,7]. However, in plants, the most frequent mechanism of miRNA regulation is direct cleavage of mRNA-target by near-perfect complementarity [8]. Several studies have reported that miRNA-guided gene regulation is essential for developmental processes [9,10] and for tolerance to biotic and abiotic stresses [11-14].

Due to the importance of miRNA in regulation of gene expression, extensive investigation aiming at the discovery of new microRNAs in several plant species has been carried out and the number of known miRNAs is increasing with over 3.300 plant-microRNA deposited sequences in the miRBase [15]. Most of these miRNAs are conserved between all plants [16]; however, there are also several miRNAs that are not. Some examples of this are: the miR403, which was identified only in eudicots, and the miR444 and miR528 that are monocots specific [17]. The process of microRNA discovery is becoming faster and an increasingly larger number of novel miRNAs are being discovered. Furthermore, analysis of the expression of miRNAs revealed that many of them are expressed only in certain tissues and/or cell types, and at specific stages of development [18-20]. According to Lu et al. (2006) [21], nonconserved miRNAs are found at low expression levels. Next generation sequence technologies have accelerated the process of small RNA discovery in many plant species and increased the recovery of rare miRNA, which together with the completion of more plant genome sequences, allowed the identification of new and weakly expressed miRNA [22].

Sugarcane is an economically important crop, mainly due to sugar and biofuel production. Sugarcane is an alogamous plant which like rice, maize and sorghum, belongs to the Poaceae family [23]. The commercial varieties belong to the genus *Saccharum* L, which consists of hybrids derived from interbreeding between *Saccharum officinarumS. sinenseS. barberiS. robustum* and *S. spontaneum* species [24]. Sugarcane has one of the most complex plant genomes, compounded by a variable ploidy number [24]. Yields of sugarcane crop can be reduced dramatically due to the influence of environmental factors such as salinity, drought, fungal and bacterial disease [25-27]. A priority for breeders is to obtain varieties with increased tolerance to abiotic and biotic stresses. Because of the reduced genetic variability in *Saccharum* hybrids, caused by recent speciation, the introduction of biotechnology tools in breeding programs can significantly contribute to the production of improved cultivars [24].

Recent research has shown that manipulation of miRNA-guided gene regulation can help in the engineering of stress-resistant-plant [28,29]. Although high throughput sequencing methods allowed for a better understanding of miRNA from non-model plants, the computational information available about sugarcane miRNA is scarce. To date, only 34 sugarcane miRNAs are deposited at the miRBase database current version (release 18). In this context, we carried out a comprehensive analysis to discover novel sugarcane miRNAs that may be regulated when subjected to drought or salt stress, and under infection of *Acidovorax avenae* ssp*. avenae*. In addition, we searched for conservation of these miRNAs in Arabidopsis, rice and maize. We discovered a total of 623 new mature miRNAs candidates in 10 sRNA libraries and identified up to 1,975 genes as potential targets for miRNA regulation in sugarcane. Our study substantially increases the number of known miRNA in sugarcane and it also provides detailed genotype-tissue-culture miRNA expression profile information for future studies.

## Results and discussion

### Computational identification of miRNAs candidates from sRNA libraries data

**Figure 1:**
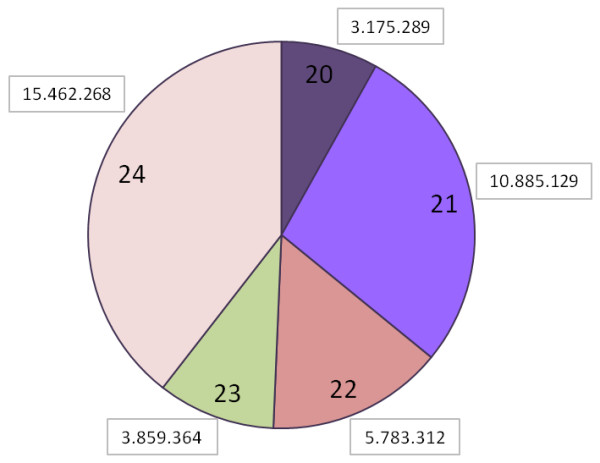
**Small RNA reads sizes distribution.** Panel depicts the length distribution of redundant small RNA dataset. In the boxes are the raw numbers of small RNA reads from each size. 21 and 24-mers were the most abundance sequences.

In order to explore the miRNA diversity in sugarcane, we constructed and sequenced 10 sRNA libraries using RNA isolated from different cultivars of sugarcane submitted to different stresses (pathogen, drought and salt). A total of 95,427,068 reads were obtained from Illumina-based sequencing, and these were used for computational identification of new miRNAs (Table 1). First, we processed raw sequence reads to remove the 3’ adaptor, “N” bases, and low complexity and sno/t/rRNA (small nuclear/transporter/ribosomal RNA) sequences, and grouped the remaining reads according to the number of unique sequences, resulting in 19,670,792 unique sequences between 20–24 nucleotides (nt) (Table 1). As shown in Figure 1, the redundant sRNA sequence size distribution after the trimming and filtering procedure showed that the most abundant sRNA in sugarcane are 21nt and 24nt in length, similar to other angiosperms [30]. The second step was to search for miRNAs candidates using miRCat pipeline, mapped to the *Sorghum bicolor* (sorghum) genome. Sorghum genome (JGI, v1.0) was used as a reference because *S. bicolor* is the most phylogenetically related species that has a genome completely sequenced (700 Mbases available) [31], while genomic resources for sugarcane consist only of 88 Mbases of EST sequences. We detected 867 miRNA unique sequences mapping to the sorghum genome, and these miRNAs were classified as known miRNAs (244 unique sequences already in the miRBase) and novel miRNAs candidates (623 unique sequences) based on BLAST search against the miRBase and PMRD (Plant microRNA database) databases (Table 1). Overall information of all and filtered reads and numbers of miRNAs for each small RNA library are summarized in Additional file 1: Table S1. Most mature miRNAs are evolutionarily conserved between plant lineages [16]. This information enabled us to computationally predict new miRNA homologs or orthologs in different plant species. After the miRBase-based classification, we selected the sugarcane miRNAs conserved in sorghum. The information of precursors that matched the criteria described in material and methods is showed in Additional file 1: Table S2. The length of miRNAs precursors ranged from 75 to 323 nt and their precursors fold-back structures have MFE (Minimum Free Energy) -20 to −174 kcal/mol. The MFEI (Minimum Fold Energy Index) is another parameter that was used to evaluate the novel miRNAs precursors. As reported previously by Zhang et al. (2006) [32], known plant miRNA precursors have MFEI value higher than others RNA (tRNA = 0.64, rRNA = 0.59 and mRNA = 0.65). In our analysis, more than 86% of new miRNAs had MFEI value higher than 0.7, suggesting that they are most to be likely miRNA precursors.

**Table 1:** Summary of results obtained after computational data mining of small RNA libraries

**Description**	**Total**	**Unique**
All reads	95,427,068	33,574,452
Filtered reads^a^	39,424,801	19,670,792
miRNAs detected^b^	2883	867
Known miRNAs^c^	1,997	244
Novel miRNAs^d^	886	623

^a^ Filtering for tRNA, rRNA, low-complexity sequence and trimming for “N” bases and 3’ adapters.

^b^ All miRNAs detected by miRCat pipeline.

^c^ Known miRNAs deposited at miRBase database.

^d^ Based on BLAST search.

**Figure 2:**
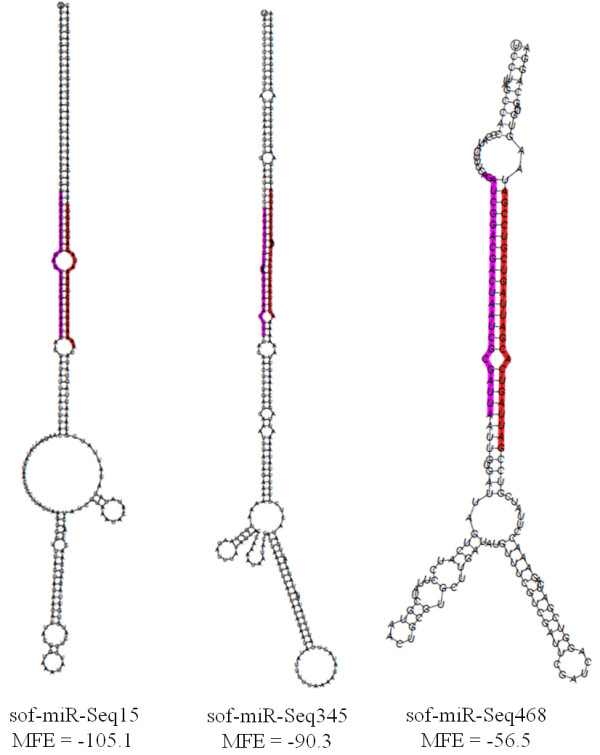
**Example of secondary structure of selected novel MIR genes, considered*bona fide*precursors.** The sugarcane mature miRNA (red), miRNA* (pink) were illustrated and MFE value of stem-loop structure were also shown.

Based on the hairpin structure profile, our sequence analysis revealed 886 precursors of novel miRNAs candidates (Table 1). After selection of these novel miRNA candidates, all 623 mature miRNAs were provisory nominated like sof-miR-Seq01. Due to the high frequency of mis-annotated miRNA, the plant small RNA research community has established a set of criteria for correct annotation of miRNA [33,34]. According to these criteria, the Class I miRNA precursors have mature miRNA and miRNA* sequence found in sRNA libraries. We identified 44 *bona fide* precursors with miRNA/miRNA* complementarity, of which 37 were unique mature miRNAs sequences. These set of microRNAs were considered high confidence and these sequences were deposited in the miRBase database. The correspondent name of these sequences in the miRBase is available in the Additional file 1: Table S3. Figure 2 shows the example of 3 precursors and their MFE values. Hairpin structures that have the lowest MFE showed the MFEI higher than 0.7; and this is strong evidence that the candidate sequence is a miRNA precursor. The structures of *bona fide* precursors are available in Additional file 1: Figure S4. However, we cannot dismiss the other miRNAs candidates that do not fit these criteria. As demonstrated by Zhang et al. (2011) [35] there are some miRNA* that are low expressed and do not necessarily appear in sequenced libraries, but the complementary mature miRNA sequences were in fact miRNA.

**Figure 3:**
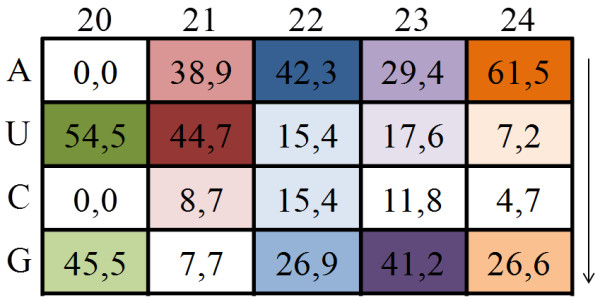
**The percentage of 5’ terminus nucleotide in relation of new miRNAs candidates lenght.** The arrow indicates the calculation of first nucleotide percentage in novel miRNAs candidates from each size. A: adenine; U: uracile (timine convertion); C: cytosine; G: guanine.

Next, we analyzed the distribution of reads by the first nucleotide at 5’end (Figure 3). Our results showed that the majority of new miRNAs candidates with 21 nt have uracile (U) at the 5’ end and the new miRNAs with 24 nt have adenine (A) in the same position. Recent studies showed distinct preference of AGO for small RNAs with a different 5’- terminal nucleotide [36,37]. Moreover, four OsAGOs that are related to AtAGO1 have conserved histidine residue in the 798 position that is critical for slicer activity of miRNA:AGO complex [38]. Analysis of small RNA sequences obtained by immunoprecipitation assays with anti-AGO1 antibodies revealed the preferential association of AGO1 with small RNAs containing 5’- terminal uridine [5]. Similar experiments with anti-AGO2 and anti-AGO4 antibodies showed an enrichment of small RNAs bearing a 5’ - terminal adenosine bound to AGO2, and AGO5 associated with small RNAs with a 5’ - terminal cytosine [5,36]. Based in the sequence similarity of the sugarcane AGO genes to those of other plant species (data not shown), it is possible that a similar nucleotide preference may exist on sugarcane, and the results in Figure 3 may indicate that the majority of the new 21 nt microRNA candidates identified in this work are canonical miRNA.

### Abundance changes of novel sugarcane miRNAs under biotic and abiotic stresses

Many studies have reported the role for miRNA in gene regulation and their involvement in responses to plant stress such as cold, salt, drought and pathogens [11-14]. In this study, we used 10 sRNA libraries generated from plants of three different experiments (Table 2).

**Table 2:** Summary of experimental procedure for sRNA libraries construction

**Assay**	**Culture condition**	**Genotype**	**Sample**	**sRNA libraries**
Water deficit^a^	soil	Tolerant - CTC15, CTC6, SP83-2847 and SP83-5073	root	Control: T0h and S0h
		Sensitive - CTC9, CTC13, SP90-1638 and SP90-3414		T24h and S24h
Salt stress^b^	*in vitro* - hydroponic	SP70-1143	leaf	Control (0 h)
				1 h; 6 h; 24 h
Pathogen infection^c^	*in vitro*	SP70-1143	whole plant	Control (CT)
				Inoculated (AA)

^a^ Drought stress induced by withholding watering.

^b^ Salt stress induced by 170 mM solution of NaCl.

^c^ Pathogen infection with *A. avenae.*

After trimming of sequence reads, miRCat pipeline and Blastn search, we identified 623 novel mature miRNAs candidate sequences in those libraries, as described above. The abundance of different miRNAs can be inferred from their frequency in the library. To compare the distribution of new miRNAs abundance in drought, salt and pathogen stresses, we normalized the miRNA reads abundance and used the electronic northern approach (methods). The read counts for miRNAs vary highly according to the kind of stress. As showed previously for soybean [39], new and known miRNAs were regulated in water deficit and pathogen assays.

Analyzing the abundance of miRNAs arising from precursor class I (high confidence miRNAs sequences) we found 26 new miRNAs assay-specific and seven miRNAs with abundance higher than 50 normalized reads counts (Table 3). miRNAs sof-miR-Seq42, sof-miR-Seq143, sof-miR-Seq488, sof-miR-Seq504, sof-miR-Seq511 and sof-miR-Seq656 were selected for experimental confirmation by stem-loop RT-PCR method. These novel miRNAs gave detectable expression levels in qRT-PCR analysis using controls samples of biotic and abiotic assays ( Additional file 1: Figure S4). Furthermore, we observed exceptionally high abundance of sof-miR-Seq513 and sof-miR-Seq513* sequences (12,593 and 8,703 reads, respectively – Additional file 1: Table S2). We confirmed the high expression of this novel miRNA and its miRNA* in saline treatment assay sample treated for 1 h ( Additional file 1: Figure S5).

**Table 3:** Electronic Northern of high confidence new miRNAs (class I) expression profiles in different libraries

**ID**	**miRNA**	miRNA*	**Eletronic Northern**									
			**Biotic stress**			**Drought stress**				**Salt stress**		
			**CT**	**AA**	**T 0h**	**T 24h**	**S 0h**	**S 24h**	**0h**	**1h**	**6h**	**24h**
sof-miR-Seq07	AAAACGTCTTATAATTTGGAG	CAAATTATAAGATGTTTTGGC	18.06	23.17	-	-	-	-	-	-	-	-
sof-miR-Seq15	AAAATTATCGTAAATAGAGGTGGC	TAGCCACTTTGAGTTACGATAATT	-	8.77	2.78	6.89	4.02	7.60	-	-	-	-
sof-miR-Seq42	AAAGTTGTGTATCTAGAAAAG	TGGCTTTTCTAGATACATAGC	67.25	37.58	-	-	-	-	2.08	-	-	-
sof-miR-Seq143	AATTCGTCGAACAGCTGCAGC	ACGCGAGCTGTTTGGCGAATT	295.17	1436.67	-	-	-	-	-	-	-	-
sof-miR-Seq151	ACAAGTTTCGTGATTTTTGGA	CGAAAATCACGAAACTTGTCG	23.04	12.53	-	-	-	-	-	-	1.24	1.12
sof-miR-Seq155	ACACATGTGGATTGAGATGAATAC	TTCACATCAATCCACATATGTTGG	15.57	27.56	-	-	-	-	-	-	-	-
sof-miR-Seq171	ACTATGTATCTAGAAAAGCTA	TTTCTAGGTACATAGCTTTTG	-	-	2.47	1.80	1.72	-	-	-	-	0.80
sof-miR-Seq183	ACTTACAGTTTGGAACGGAGG	TCTGTTCCAAATTGTAAGTCG	-	5.64	-	-	-	-	-	-	-	-
sof-miR-Seq253	AGTCCCGAAACCTTAGTCCCGGCT	GAACCGGGACTAAAGGTGGGACAT	21.80	6.26	-	-	-	-	-	6.22	-	5.93
sof-miR-Seq313	ATGCCTTATAATTTGGGATGGAGA	CTCCATCCTAAATTATAAGACATT	-	-	-	-	-	-	-	-	0.77	-
sof-miR-Seq345	ATTTGACTGACACGGATTCTAGGA	TAGAGTTGTCCTAAGTCAAACTTT	3.74	-	-	-	-	-	-	-	-	-
sof-miR-Seq375	CCGGGGCCAGATCTCAGAAGC	TTCTGACTTCTGGCCCCTGCT	-	-	-	-	-	-	-	-	-	2.24
sof-miR-Seq376	CCTGTTTGGATCAGCCAAGGC	CTAGCTGATCCAAACAGGCCC	13.08	9.39	-	-	-	-	-	-	-	-
sof-miR-Seq393	CTAGCATGTTCCTCCTAAGAG	TTCTTGGGAGGAGCATGCTAG	-	5.01	-	-	-	-	-	-	-	-
sof-miR-Seq394	CTCCGTCCTAATATATAAGGC	CTTATATACTAGGACGGAGGG	4.36	-	-	-	-	-	-	-	-	-
sof-miR-Seq409	GAAACGAATCTTTTAAGTCTAATT	AACTAGACTCAAAAGATTCATCTC	3.11	-	-	-	-	-	-	-	-	-
sof-miR-Seq468	GATTAGTCACGATTAGTCGTCCGA	AGATCGGACGACTAATCGCGATTA	-	-	-	-	-	-	-	-	0.77	-
sof-miR-Seq488	GCGTGCAAGGAGCCAAGCATG	TGCCTGGCTCCCTGTATGCCA	-	-	572.49	482.28	497.41	570.24	-	-	-	-
sof-miR-Seq501	GGCATGGGAACATGTAGGAAGG	TTCCTGATGCCTCCCATGCCTA	11.21	-	-	-	-	-	-	-	-	-
sof-miR-Seq504	GGGAGCAATTCGTCGAACAGC	AGCTGTTCGACGAATGCCTCC	123.30	-	-	-	-	-	-	-	-	-
sof-miR-Seq509	GGGCCCAAATAGCAAGTGTTGTGA	CTCACAACACTTGCTATTTGGG	-	-	-	-	-	-	1.48	-	2.01	0.96
sof-miR-Seq511	GGGCGGTCACCTTGGCTAGC	TAGCCAAGGATGACTTGCCT	132.64	-	-	-	-	-	-	-	26.48	-
sof-miR-Seq513	GGGGGCGGACTGGGAACACAT	TGTGTTCTCAGGTCGCCCCCG	-	-	-	-	-	-	-	3404.36	-	-
sof-miR-Seq536	GTGCGGTTCTCCTCTGGCATG	TGCCAAAGGAGAATTGCCCTG	-	-	-	-	-	-	1.93	-	-	-
sof-miR-Seq538	GTGGCAGTAGAATTAATGAAGGGA	TTCTATCTCTATTAATTGTGTTGC	-	12.53	-	-	-	-	-	1.62	-	-
sof-miR-Seq565	GTTTTTCTCGCCGGGTGAGAAGGC	ATTCTCACTTGGGCGACGGAAAGG	-	3.13	-	-	-	-	-	-	-	-
sof-miR-Seq568	TAACAAGTTTAGGGATCTAGA	TTTTGGGTCCCTAAACTTGTT	-	-	2.47	3.30	-	-	-	-	-	0.80
sof-miR-Seq587	TAGCATGTTCCTCCTAAGAGC	TTCTTGGGAGGAGCATGCTAG	18.68	-	-	-	-	-	-	-	-	-
sof-miR-Seq594	TATCTAGAAAAGCTAAAACGT	GATGTTTTGGGTTTTCTAGAT	-	-	-	-	-	-	0.74	-	-	-
sof-miR-Seq598	TATTTGTGGACTCATGGACAT	GTCCGTGAGTCCACAAATAGG	3.11	3.13	-	-	-	-	1.78	-	2.48	1.76
sof-miR-Seq610	TCCATTCCAAATTGTAAGATG	GTCTTATAATTTGGAATGGAG	30.51	6.89	-	-	-	-	-	-	-	-
sof-miR-Seq648	TGGATGTACCAAAAAAGTCAAAGC	GTCGCTTTGACTTTTTTGGTACAT	-	-	-	-	-	-	-	3.24	-	-
sof-miR-Seq656	TGGGCGGTCACCTTGGCTAGC	TAGCCAAGGATGACTTGCCTA	-	65.13	27.51	38.64	72.99	62.81	-	-	-	-
sof-miR-Seq670	TGTTGAGGCTGGAGCGAAACTCGG	CAAGTTTGGTTTTGGTAATTAATG	-	12.53	-	-	-	-	-	-	-	-
sof-miR-Seq678	TTAGCGTCAAGAGACGAACACACT	AAGTGTGTTCCTCTATTTGACGCT	-	3.76	-	-	-	-	4.74	6.49	2.79	7.53
sof-miR-Seq700	TTGTGAGAGAAAAATACTGTTGGC	AACGAACAGTATTTTTCTCTTACA	-	16.28	-	-	-	-	-	-	-	-
sof-miR-Seq720	TTTTTGGTACATTGAATTTGC	AATTCGATGTACCAAAAAAGT	-	11.27	-	-	-	-	-	-	-	-

ID: identification of sequences; CT: non inoculated plants; AA: under *A. avenae* infection; T 0 h and T 24 h: tolerant genotypes in control and drought stress conditions, respectively; S 0 h and S 24 h: sensitive genotypes in control and drought stress conditions, respectively; 0 h: plant without salt stress; 1 h, 6 h, 24 h: plants after salt treatment

The number of mature miRNA reads were normalized by transcripts *per* million. The mature miRNA and miRNA* sequences were also shown.

**Figure 4:**
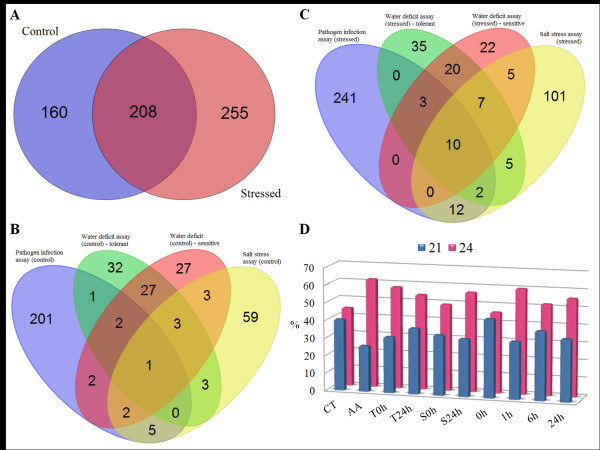
**Global distribution analyses of new mature miRNAs candidates.** Venn diagram comprising the new miRNAs candidates distribution in each condition: **A)** Comparison of all control and all stresses libraries; miRNAs distribution in control sRNA libraries **(B)**, and pathogen infection, drought and salt stressed libraries **(C)**; **D)** Distribution of canonical and long-miRNAs in each libraries. CT: non inoculated plants; AA: under *A. avenae* infection; T 0 h and T 24 h: tolerant genotypes in control and drought stress conditions, respectively; S 0 h and S 24 h: sensitive genotypes in control and drought stress conditions, respectively; 0 h: plant without salt stress;1 h, 6 h, 24 h: plants after salt treatment.

The analysis of miRNAs arising from all precursor classes revealed differential accumulation of certain new miRNA in the context of a particular stress ( Additional file 1: Table S6). Only sof-miR-Seq296 was induced constitutively in all libraries (control and treated). The biotic stress library showed higher exclusive expression of new miRNAs (155 sequences). Figure 4A shows the distribution of the 623 novel sugarcane miRNAs found in either treatment (255) or control samples (160) or in both (208). Because the control libraries were constructed with three types of tissues of different genotypes cultivated *in vitro*, hydroponic and soil condition, we analyzed the new miRNAs distribution in all control conditions (Figure 4B). Only one novel miRNA candidate were shared between all control libraries. In control plants of pathogen infection assay (*in vitro* cultivation) were found specifically 201 new miRNAs candidates and in control plants of salt stress assay (hydroponic cultivation) 59 were found. Control plants of the water deficit assay (tolerant and sensitive) shared 27 new miRNAs, in contrast with control plants of the pathogen infection and salt stress assays that are from the same genotype and had five new miRNAs in common. These results showed that plants grown under the same condition (plants of water deficit assay grown in soil), independently of their genotype, share similar numbers of detectable new miRNA. Many genome regions are silenced through DNA methylation, histone modifications and small RNA-direct DNA methylation, while others are activated by the up- or down-regulation of the same epigenetic mechanisms [40,41]. These modifications in chromatin structure may be activating much of the new miRNAs detected in the present study. Interestingly, plants of the same genotype (SP70-1143) firstly grown *in vitro* followed by cultivation in a hydroponic system (control plants of salt stress assay), do not show a higher number of novel miRNAs candidates (76 against 214 in control plants of pathogen infection assay). Nevertheless, these control plants showed higher amount of new miRNAs than control plants of water deficit assay (tolerant and sensitive genotypes). In general, the new miRNAs expression in control plants seems to be genotype-tissue-culture dependent.

In order to find new sugarcane miRNAs that may be involved in the regulation of the plant’s responses to stress we investigated the distribution of miRNAs sequences candidates in the sRNA libraries from treated samples (Figure 4C). The number of shared new miRNAs was increased (1 to 10) in all stressed libraries in contrast to all control libraries (Figure 4B and C). Plants under pathogen infection showed the highest number of novel miRNA (268). In the drought stress, tolerant genotypes showed an increase of the new miRNAs number (68 to 82) and sensitive genotypes remained unchanged. However, in sensitive genotypes under drought stress the number of exclusive new miRNAs decreased weakly (27 to 22). In addition, we observed a high induction of novel exclusive miRNAs candidates in the salt stress libraries, where we identified twice as many novel miRNA expressed under the stress condition than in the control samples (59 to 101).

Plants contain a complex network of small RNA pathways. The canonical pri-miRNA is cleaved by DCL1 and results in mature miRNA 21nt in size. However, some researches described a novel class of *bona fide* miRNA [7,42]. This class was denominated long-miRNA (24 nt length) and their precursors are processed by DCL3 as well as siRNA. Contrary to siRNAs that require PolIV and RDR2 to be processed, stem-loop precursors of long-miRNA are originated from PolII and do not require RDR2 [7]. The other characteristic of long-miRNA is the mechanism of regulation. Recent study in rice described that long-miRNA mediated DNA methylation by AGO4 [7]. Canonical miRNAs and long-miRNAs comprised more than 91 % of all miRNAs species characterized in this study. We detected different distribution of those species in stresses libraries. In biotic stress we observed a reduction of 21-nt-species and increase in 24-nt-species, similar to salt stress and drought stress sensitive genotypes libraries (Figure 4D). Instead, drought stress tolerant genotypes libraries showed an increase of 21-nt-species and a reduction of 24-nt-species. These analyses suggest that biotic, salt stress and drought stress (in the case of sensitive genotypes) may be regulating pathways by miRNAs from the same species (24-nt).

### Target prediction and function analysis

In plants, miRNA regulate gene expression by either cleavage of mRNA or translation repression targets [10,43]. In addition, it has been described recently that some miRNA may regulate gene expression by DNA methylation [7]. Computational identification of plant miRNA-target is possible due the perfect or near-perfect match between miRNA/target in complementary sites [44]. Using two published methods [45,46] we predicted miRNA targets. We first searched for targets of new miRNAs candidates in a sugarcane database (*Saccharum officinarum* ESTs – DFCI gene index) and then in the sorghum databases (*S. bicolor* gene models and ESTs – DFCI gene index). The results of these analyses are available in Additional file 1: Table S7 and Additional file 1: Table S8. We identified 1,975 putative target genes for 483 new miRNAs, with an average of 4 targets per miRNA candidate. In sorghum, the average of gene targets per miRNA was lower than sugarcane (2.4), showing 895 targets for 373 miRNA. Importantly, the results demonstrated that most sugarcane targets have conserved miRNA candidate recognition sites as they are found in sorghum as well as in sugarcane.

Due the importance of miRNA in regulating a variety of biological processes, we analyzed the putative miRNA targets in order to understand the biological function of novel miRNAs candidates. Analysis of the sugarcane Tentative Consensus (TC) EST assembly discovered 776 new miRNAs candidates targets that had versatile functional annotation and 336 were of unknown function. MADS-box transcription factor MADS2 and 60 S acidic ribosomal protein P2B (RPP2B) were overrepresented. MADS2 is a transcription factor that regulates development [47,48] and RPP2B plays an important role in disease resistance to bacterial pathogens [49]. The expression of MADS2 was verified using qRT-PCR and an induced profile was shown after 1 h of saline treatment related to control ( Additional file 1: Figure S9). This result suggests a regulation of MADS2 by sof-miR-Seq09 and sof-miR-Seq19 where the new miRNAs candidates were repressed after 1 h of saline treatment.

Most targets annotated as MADS2 were recognized by 21nt miRNA candidate. Similarly, the majority of microRNAs conserved among species target transcription factors [50,51], however, the majority of new sugarcane miRNAs candidates that regulate RPP2B have 24 nt in length. According to Wu et al. (2010) [7], long-miRNA may direct DNA methylation around their recognition site in the target loci. This type of miRNA may act in their own precursors *in cis* and also in their targets *in trans* to guide DNA methylation [52]. Because of the features applied on the search of complementary sites of miRNA and mRNA, we have also identified targets that were possibly regulated by DNA methylation. In sugarcane, 140 new miRNAs candidates did not have predicted targets, of which 113 have 24 nt length. The sugarcane genome sequence is not available, we cannot exclude the possibility that *bona fide* targets may exist and they have been not sequenced yet. Another possibility is that these miRNAs are targeting their own loci *in cis*, and that is why we did not detect their targets by the pipeline used. Interestingly, when we searched targets of these novel miRNAs candidates in sorghum, a similar profile was observed and the higher amount of miRNAs candidates that did not have predicted targets were 24-nt-species (190 in 250 new miRNAs candidates).

The sugarcane targets regulated by high confidence new miRNAs arising from the precursor class I were listed in Table 4, and the conserved miRNA targets sites in sorghum are highlighted. Thirty three new miRNAs had targets in sugarcane and 29 in sorghum. Targets annotated, as serine/threonine kinase, Myb protein, MADS-box, zinc finger protein-like were potentially regulated by different new miRNAs class I. In this set, targets annotated like AMP-binding protein were overrepresented. This is a defense related protein that is involved in the regulation of defense response [53].

**Table 4:** Predicted sugarcane and sorghum mRNA targets for the high confidence novel miRNAs

**miRNA ID**	**Sugarcane targets**^**a**^	**Sorghum targets**^b^
sof-miR-Seq7	TC130098,TC151912,TC148492,TC113248,TC152641,TC150124,TC132416,TC114872,TC140938,TC115982,TC120001	+
sof-miR-Seq15	No TC found	+
sof-miR-Seq42	TC153375,TC149921,TC131074,TC139079	+
sof-miR-Seq143	No TC found	+
sof-miR-Seq151	TC153350,TC116972,TC137677	+
sof-miR-Seq155	TC152159,TC125281,TC149817,TC136354	+
sof-miR-Seq171	TC149921,TC132416,TC152095,TC148248	+
sof-miR-Seq183	TC149944,TC132416	+
sof-miR-Seq253	No target found	+
sof-miR-Seq313	TC154059,TC139862,TC140501	+
sof-miR-Seq345	No TC found	-
sof-miR-Seq375	TC136870,TC153496,TC134975	+
sof-miR-Seq376	No TC found	-
sof-miR-Seq393	TC152280,TC116268	+
sof-miR-Seq394	No TC found	-
sof-miR-Seq409	TC129500,TC154465,TC152078,TC154496,TC150491,TC154742,TC131475,TC116420,TC142998,TC146859,TC134345,TC133965,TC153566,TC149558,TC149819,TC154535,TC150277	+
	TC129500,TC154465,TC152078,TC154496,TC150491,TC154742,TC131475,TC116420,TC142998,TC146859,TC134345,TC133965,TC153566,TC149558,TC149819,TC154535,TC150277	
sof-miR-Seq468	TC153570,TC141767	+
sof-miR-Seq488	TC152925	-
sof-miR-Seq501	TC151370	-
sof-miR-Seq504	TC126404	+
sof-miR-Seq509	No target found	+
sof-miR-Seq511	TC124067,TC118309	+
sof-miR-Seq513	TC141387	-
sof-miR-Seq536	TC139920,TC130151,TC120175	+
sof-miR-Seq538	No TC found	-
sof-miR-Seq565	No TC found	-
sof-miR-Seq568	No target found	+
sof-miR-Seq587	TC139519,TC152280	+
sof-miR-Seq594	TC115982,TC149921,TC113248,TC140938,TC132416,TC148248,TC140476	+
sof-miR-Seq598	TC124210	+
sof-miR-Seq610	TC122224,TC140470,TC145127,TC142562,TC120001,TC150124	+
sof-miR-Seq648	TC137080,TC134144,TC117196	+
sof-miR-Seq656	No TC found	+
sof-miR-Seq670	TC138461,TC153815,TC149322,TC146441,TC153901,TC142328,TC154437,TC154455,TC125410	+
sof-miR-Seq678	TC133894,TC129132	+
sof-miR-Seq700	TC152242,TC146024,TC117281,TC152372,TC146587,TC153355,TC154348,TC148421,TC128182,TC148979,TC120001,TC117481,TC134335,TC113000,TC138157,TC130788,TC129900	+
sof-miR-Seq720	TC125467,TC129883,TC145851,TC144060	+

^a^ Identification of sugarcane targets. miRNAs that only have singletons as targets were identified by No TC found. MiRNAs that did not have predicted targets were identified by No target found.

^b^ Plus and minus signals indicate presence or absence (respectively) of sorghum genes containing conserved miRNA recognition sites.

**Figure 5:**
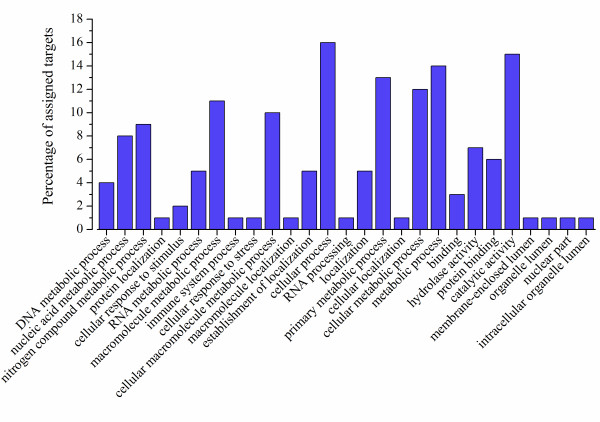
**Gene Ontology enriched terms of novel miRNA targets.** Bars indicate the percentage of total annotated sorghum and sugarcane targets genes mapping to GO terms. Only putative targets arising new miRNAs class I were shown.

Next, sugarcane and sorghum identified targets were subjected to gene ontology analysis. We extracted the unique IDs of targets and compared them with GO annotations of *S.bicolor* gene models, and *S.bicolor* and sugarcane TC annotations. The GO numbers of targets were subjected to agriGO toolkit [54]. Among the distribution of GO annotation of the targets, only miRNA class I targets were represented in Figure 5. The most representative GO was the metabolic process (46,6%). The enrichment of this GO may be consistent with the fact that six libraries of sRNA were constructed from plants cultivated *in vitro*, which may have had their development accelerated by combination of plant hormones [55]. In addition, genes involved in immune system processes and cellular responses to stresses were present there, presumably due to the stress treatments.

### Conservation of novel miRNAs in diverse plant species

Flowering plants comprise approximately 250,000 species and originated around 200 million years ago [56]. Phylogenetic analyses have recently resolved major relationships among angiosperm group using both molecular and morphological information [57,58]. The wide variability of the angiosperms allow their adaptation to diverse environmental conditions and also their domestication [59]. The estimate of divergence time, acquired using plastid exons and rDNA revealed that monocots and eudicots diverged about 150 million years ago [56]. Within monocots; rice, maize, sugarcane and sorghum (Poaceae family) originated approximately 60 million years ago [60]. Early in the radiation of eudicots plants, the Arabidopsis family (Brassicaceaea) diverged (~85 million years ago) [56].

**Figure 6:**
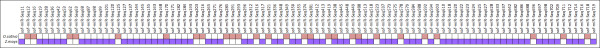
**Partial conservation of new sugarcane miRNAs candidates between rice and maize.** Boxes are highlighting the presence of new miRNA precursor identified by miRCat pipeline in theses plant species.

Phylogenetic conservation analysis of novel miRNAs between closely related species provides supporting evidence and has been used to annotate new miRNAs [61]. Based on miRCat pipeline we searched for the precursors of the novel sugarcane mature miRNAs candidates in two monocots (*Oryza sativa* and *Zea mays*) and Arabidopsis. We did not identified conserved precursors of new sugarcane miRNAs candidates in Arabidopsis. In rice, we identified 27 homologs of these mature miRNAs, while in maize, the closest relative to sorghum and sugarcane of the three reference species, we identified phylogenetic evidence for 69 novel miRNAs (Figure 6).

In polyploid genomes, genetic and epigenetic modifications can quickly change the structure and function of genomes [62]. Vincentz et al. (2004) [59], using Arabidopsis and rice genomes and sugarcane transcriptome, showed that some genes are monocot-specific, diverging from eudicots. The most accepted hypothesis for miRNA origin is duplication of their target genes, auto-, tandem or segmental duplication [63]. They are fast-evolving sequences that may present a divergence degree reflecting the phylogenetic divergence between species [63]. As mentioned above, the most phylogenetic related species showed more conserved miRNA (Figure 6). However, since the rate of evolution is different between species, many miRNA are not conserved and are, therefore, species-specific. The data suggest that miRNA evolution is on-going process and some of miRNA may be originated in a species during plant evolution.

## Conclusions

We have shown that next-generation sequencing technologies have a substantial impact on miRNA discovery of non-model plants. In our study, numerous small RNA libraries were constructed from sugarcane plants submitted to drought and salt stress, or to pathogen infection. By using bioinformatics analysis, we detected a large number of new sugarcane miRNAs candidate sequences and inferred about their possible biological importance analyzing their deep profiling in different genotypes, tissues and conditions, and also through the analysis of their putative target genes. As regulatory molecules with ancient origin, new sugarcane miRNAs shared greater homology with their monocot-related species, indicating that miRNA are fast-evolving sequences whose origin is closely related with plant evolution. Our findings provide the first large scale miRNA discovery in sugarcane and help to clarify about potential miRNA roles in regulatory pathways of this and other crops.

## Methods

### Plant material and experimental procedure

#### Water deficit assay

Stalks of sugarcane cultivars, with different drought sensitivities, were provided by the Centro de Tecnologia Canavieira (CTC). Based on chlorophyll and water content measurements, cultivars CTC15, CTC6, SP83-2847 and SP83-5073 and CTC9, CTC13, SP90-1638 and SP90-3414 are considered as drought tolerant and sensitive, respectively. Stalks were germinated and grown in 5 L pots in a greenhouse at 28°C. After three months, the plants were exposed to drought stress by withholding watering. Treated and control roots were harvested at 0 and 24 hours of treatment, respectively. Four small RNA libraries for deep sequencing were constructed from RNA pools of sensitive and tolerant sugarcane cultivars submitted to drought stress and control plants.

#### Salt stress assay

*In vitro*-grown sugarcane plantlets (*Saccharum sp*. Cv SP70-1143) were rooted in Murashige and Skoog media supplemented with sucrose (2%), citric acid (150 mg/L), kinetin (0,1 mg/L), and IBA (0,2 mg/L). Plants were maintained at 110 mE m-2 s- luminosity, 12 h photoperiod, at 28°C. After the development of a root system (nearly a month) plantlets were transferred to hydroponic system compound in plastic containers (16 liters) supplemented with Hoagland solution [64]. Plantlets were acclimated during two weeks in a greenhouse at 28°C and then NaCl 170 mM solution was added. Control plants were maintained in distillated water. Leaves of treated and control plantlets were harvested at 0, 1, 6 and 24 hours after treatment. A set of five plants was collected for each time point of the experiment, and the pooled material was used in the construction of four small RNA libraries.

#### Pathogen infection assay

*Acidovorax avenae subsp avenae* obtained from the Culture Collection of the Instituto Biológico (IBSBF) was grown in NA medium (beef extract 3 g/L; Peptone 5 g/L; NaCl 5 g/L) at 28°C. *In vitro*-grow sugarcane plantlets were cultivated as described in the saline stress experiment. After the development of a root system, vigorous and pathogen-free plants were divided in two halves with a scalpel. One half was inoculated immersing the root system for 5 minute in a suspension of *A. avenae* in distilled water (106 CFU mL-1) and then washed twice with distilled water in order to eliminate superficial bacteria. The other half was used as control, immersing the root system in distilled water for 5 minutes and then washed twice with distilled water. Inoculated and control plants were transferred to MS media and kept for seven days. After this period, whole plants were harvested and examined for bacterial colonization by plate counting (data not shown), and small RNA libraries of control and inoculated plants were constructed.

### RNA extraction and sequencing small RNA library construction

Total RNA was isolated from fresh root, leaves and whole plants materials using Trizol (Invitrogen, CA, USA) as described by the manufacturer. The amount of RNA was measured using Thermo Scientific NanoDrop™ 2000c Spectrophotometer and then quality was verified by electrophoresis on a 1% agarose gel. Total RNA (~10 μg) from ten samples (4-water deficit assay, 4-saline stress assay and 2-pathogen assay) was sent to Fasteris Life Sciences SA (Plan-les-Ouates, Switzerland) for construction of small RNA libraries and subsequent sequencing of 20–30 nt single-end reads. Quality of the sequences was evaluated by measuring the quality of the reads according to the percentage of bases having a base quality greater or equal than 30 (Q30). On average, 80% of the channel had Q30 of quality. After this, 3’ Illumina adapters (CTGTAGGCACCATCAAT) and “N” bases were trimmed of the reads and reads in between a 20–24 nt range were separated for further analysis.

### Prediction of new miRNA candidates

Small RNA reads trimmed were filtered out if they had an exact full-length match to known plant tRNA or rRNA sequences and low-complexity sequences. Using the UEA sRNA toolkit-Plant version filter pipeline (http://srna-tools.cmp.uea.ac.uk/) [65], and three different databases - all RNAs from Rfam (excepted miRNAs), all Arabidopsis tRNAs from The Genomic tRNA Database and all plant t/rRNA sequences from EMBL release - reads with low-complexity (less than 3 different bases) and both sense and antisense, matches with different types of RNAs (e.g. sno/t/rRNAs) were removed. The remained reads were then submitted to the UEA sRNA toolkit-Plant version miRCat pipeline to predict miRNAs from high-throughput small RNA sequencing data. miRCat was running with minimum sRNA abundance of 5 reads, minimum size of 20 nt, maximum size of 24 nt and maximum number of 16 genome hits. Sequences of sRNA were then mapped to a *Sorghum bicolor* genome (JGI, v1.0) to find clusters of sRNA that matched the following criteria: i) clusters must contain no more than four non-overlapping sRNAs; ii) each sRNA in a cluster must be no more than 200 nt from its neighbor; iii) at least 90 % of sRNAs in a cluster must be in the same orientation. The most abundant sRNA read within a cluster is chosen as the likely miRNA candidate. The flanking sequence surrounding this sRNA is extracted from the genome using a 75 nt of window length. Each sequence window is then folded using RNAfold. miRCat trims and analyses the resulting secondary structure to verify whether it forms a characteristic miRNA hairpin and executes the following additional checks: i) no more than 3 consecutive mismatches between miRNA and miRNA*; ii) at least 17 of the 25 nucleotides centered around the miRNA must be involved in base-pairing; iii) the hairpin must be at least 75nt in length; iv) at least 50 % of bases in the hairpin should be paired. The most stable valid hairpin from each of the sequence windows is then chosen as the precursor miRNA candidate. The precursor miRNA candidate is then tested using randfold (using a cutoff of 0.1). Additional minimal folding free energy index (MFEI) was calculated manually according to Zhang et al (2006) [32]. The miRNAs candidates were searched against miRBase database release 17 (http://www.mirbase.org/ftp.shtml) and PMRD: Plant microRNA database (http://bioinformatics.cau.edu.cn/PMRD/) using standalone BLAST [66] blastn with default parameters. Only reads with e-value >10^-3^ at miRBase were considered with new sugarcane miRNAs candidates. The folding structures of the precursors of the new miRNA with miRNA* identified were carried out with UEA sRNA toolkit-RNA hairpin folding and annotation tool, that uses the Vienna Package to obtain the secondary structure of a precursor sequence and highlighting up miRNA/miRNA* sequences on hairpin structure. These set were considered as high confidence miRNAs sequences and these sequence were deposited in the miRBase database.

The analysis of the conserved novel sugarcane miRNAs in Arabidopsis, rice and maize was carried out following the miRCat pipeline using new miRNAs mapped to the Arabidopsis (*Arabidopsis thaliana*, TAIR v.9), rice (*Oryza sativa*, TIGR/MSU v5.0) and maize (*Zea mays*, AGP v1.0) genomes, respectively.

### Identification of potential miRNA targets

The high degree of homology between the mature sequence of the miRNA and the cleavage site in their targets allows *in silico* identification of putative miRNA targets in the databases. To identify the putative new miRNA targets we used the standalone-based UEA sRNA toolkit-Plant target prediction pipeline. The standalone version of the plant target prediction tool permits one to choose transcripts databases for searching targets. In this investigation we used three different transcripts databases: *S. bicolor* gene models [31], *S. bicolor* ESTs – DFCI gene index release 9 and *Saccharum officinarum* ESTs – DFCI gene Index release 3. The rules used for the plant target prediction are based on criteria previously suggested by other researchers [45,46]. miRNA/target duplexes must obey the following rules: i) no more than four mismatches between sRNA and target (G-U bases count as 0.5 mismatches); ii) no more than two adjacent mismatches in the miRNA/target duplex; iii) no adjacent mismatches in positions 2–12 of the miRNA/target duplex (5' of miRNA); iii) no mismatches in positions 10–11 of miRNA/target duplex; no more than 2.5 mismatches in positions 1–12 of the of the miRNA/target duplex; iv) and Minimum free energy (MFE) of the miRNA/target duplex should be > = 74% of the MFE of the miRNA bound to its perfect complement. All putative targets regulated by new sugarcane miRNAs were subjected to gene ontology analysis. We extracted the unique IDs of targets and compared them with GO annotations of *S. bicolor* gene models (available at http://genome.jgi.doe.gov/Sorbi1/Sorbi1.download.ftp.html), *S. bicolor* TC annotations (available at http://compbio.dfci.harvard.edu/cgi-bin/tgi/tc_ann.pl?gudb=sorghum) and *Saccharum officinarum* TC annotations (available at http://compbio.dfci.harvard.edu/cgi-bin/tgi/tc_ann.pl?gudb=s_officinarum). The GO numbers of targets were subjected to agriGO [54]. The singular enrichment analysis (SEA) was performed to find enriched GO terms within annotated miRNA targets.

### Validation of miRNA expression and target by qRT-PCR

The expression profiles of nine sugarcane’s new mature miRNAs were assayed by stem–loop reverse transcription-PCR [67,68]. Total RNA extracted from leaves, roots and whole plants using in the small RNA libraries construction was treated with DNaseI (Promega). Total RNA is then reverse transcribed into cDNA using Super-ScriptIII reverse transcriptase (Invitrogen) using in the same reaction RT primers specific for all miRNAs analyzed ( Additional file 1: Figure S5). To analyze the expression profile of mature miRNA and MADS2 target, qRT-PCR was used with SYBR Green PCR Master Mix (Applied Biosystems). To each well, 1 μL of first strand cDNA, 5 μL of SYBR Green solution, 2 μL of the forward primer (10 μM) and 2 μL of reverse primer (10 μM) were added ( Additional file 1: Figure S4). U6 and GAPDH were used as the internal control of miRNA and MADS2 expression, respectively. qRT-PCR was performed using Applied Biosystems 7500 Real-Time PCR Systems.

## Competing interests

The authors declare that they have no competing interests.

## Authors’ contributions

FT, CG, MC-B and CAR authors contributed equally to this work; FT and CG wrote the manuscript and carried-out most of bioinformatics analyzes; MC-B performed real-time quantification RT-PCR; FT, MC-B and CAR carried-out the plant experiments and RNA extraction; PCGF and ASH coordinated the study; MT, RAM and LF participated in the libraries construction and sequencing; MT and PCGF critically revised the article. All authors read and approved the final manuscript.

## Supplementary Material

Additional file 1 **Table S1.** Summary of results obtained after computational data mining for each small RNA library**.** Overall information of all and filtered reads, numbers of miRNAs detected, known and novel miRNAs candidates. **Table S2.** miRCat analyses results of new miRNAs precursors. Overall information of precursors that matched to the criteria for miRNA prediction. **Table S3**. Correspondence among names of high confidence new microRNAs. List of names used in this paper, and the new names of microRNAs after deposit in the miRBase. **Figure S4.** Predicted precursor structure of high confidence novel miRNAs (class I) identified. The sugarcane mature miRNA (red), miRNA* (pink) were illustrated in pre-miRNA with chromosome and locus information based in sorghum genome. All novel miRNAs sequences were denominated sof-miR-Seq following the number. **Figure S5.** Confirmation of new miRNA expression. Validation by stem-loop RT-PCR of eight new miRNAs chosen from our libraries. Primers used were listed in the table. Z. **Table S6.** Electronic Northern of all new miRNAs candidates in each library. The mature new miRNA reads were normalized to transcripts *per* million. **Table S7.** Putative targets of all new miRNAs candidates in sugarcane. EST sugarcane data from Gene Index version 3.0 was used to search for potential new miRNA targets by UEA sRNA toolkit-Plant target prediction pipeline. **Table S8.** Putative targets of all new miRNAs candidates in sorghum. EST sorghum data from Gene Index version 9.0 and Sorghum gene models (JGI v.1.0) were used to search for potential new miRNA targets by UEA sRNA toolkit-Plant target prediction pipeline. **Figure S9.** Relative expression profile of MADS Box. Relative expression of putative targets of sof-miR-seq09 and sof-miR-seq19 estimated within control and 1 h after saline treatment. Click here for file
